# Effect of temperature on the development and survival of immature stages of the carambola fruit fly, Bactrocera carambolae, and the Asian papaya fruit fly, Bactrocera papayae, reared on guava diet

**DOI:** 10.1093/jis/14.1.126

**Published:** 2014-09-15

**Authors:** Solomon Danjuma, Narit Thaochan, Surakrai Permkam, Chutamas Satasook

**Affiliations:** 1 Department of Crop Production, Faculty of Agriculture, Ibrahim Badamasi Babangida University, P.M.B 11, Lapai, Niger State, Nigeria; 2 Department of Pest Management, Faculty of Natural Resources, Prince of Songkla University, Hat Yai, Thailand 90112; 3 Current address: Department of Biology, Faculty of Science, Prince of Songkla University, Hat Yai, Thailand 90112

**Keywords:** Tephritidae, thermal constant, linear model, peninsular Thailand

## Abstract

Members of the *Bactrocera dorsalis* Hendel (Diptera: Tephritidae) complex constitute well-recognized destructive pests of fruits in peninsular Thailand. The development and survival of immature stages of the carambola fruit fly, *Bactrocera carambolae* Drew & Hancock, and the Asian papaya fruit fly, *Bactrocera papayae* Drew & Hancock *,* were compared at six constant temperatures of 15, 20, 25, 27, 30, and 35°C, 70 ± 5% relative humidity, and a photoperiod of 12:12 (L:D). The objectives were to determine the effect of temperature on the developmental stages for optimizing rearing and to understand the geographical pattern of occurrence of these fruit fly species. A strong and positive linear relationship was observed between temperature and developmental rate of immature stages of *B. carambolae.* Similarly, a strong and positive linear relationship was observed between temperature and developmental rate of *B. papayae.* A temperature summation model was used to estimate the lower threshold temperature and the thermal constant. *Bactrocera papayae* was significantly faster in development and higher in survival and appeared to be better adapted to low temperatures than *B. carambolae,* as it exhibited the lowest threshold temperatures at all immature stages. The observed differences in response to various temperatures revealed to some extent the impact of temperature on these species’ distribution in peninsular Thailand and other parts of the world.

## Introduction

The genus *Bactrocera* Macquart (Diptera: Tephritidae) is recognized worldwide for its destructive impact on agriculture. Besides causing billions of dollars in direct losses to a wide variety of fruit, vegetables, and flower crops (e.g., citrus, apple, mango, sunflower), which limits the development of agriculture in many countries due to reduction in farm income, it also leads to overuse of pesticides. Growers and governments face rising costs as they attempt to meet demands for food. Therefore, pest-free or low-pest density zones are being advocated worldwide for fruit exports with minimal or zero quarantine restrictions ( Carroll et al. 2004 , FAO 2006). The damage, if uncontrolled, may result in a total loss of the crop in question ( Yong et al. 2010 ). The genus *Bactrocera* is known to be largely endemic to Asia and the Pacific. Among the serious pest species, several are indigenous to peninsular Thailand and Malaysia and are members of the *Bactrocera dorsalis* Hendel complex, including *B. dorsalis* sensu stricto Hendel, *Bactrocera carambolae* Drew & Hancock, *Bactrocera papayae* Drew & Hancock, and the cucurbit feeders *Bactrocera cucurbitae* (Coquillet) and *Bactrocera tau* (Walker) ( Drew and Hancock 1994 , Clarke et al. 2001 ).

*Bactrocera carambolae* and *B. papayae* are distributed widely in Southern Thailand, where they affect different kinds of fruits and vegetables. Drew8yt and Hancock (1994), Ranganath and Veenakumari (1995) , Allwood et al. (1999) , and van Sauers-Muller (2005) worked extensively on the host plant records for fruit flies (Diptera: Tephritidae) in Southeast Asia. Their work revealed that *B. carambolae* and *B. papayae* are polyphagous species of tephritid flies found in Southeast Asia. They reported 76 and 193 host species for *B. carambolae* and *B. papayae,* respectively, in this region. Among the listed hosts, guava was found to be the most infested compared with any other host species listed.

Guava is one of the most ubiquitous fruits appearing at all stalls and markets in Thailand. It is an important source of income and represents an important part of the gastronomic culture for Thai people ( Victor 2009 ). The fruit is produced at small scale and sometimes even at subsistence-level farming. For several tropical fruits, the production is mainly by smallholder producers and largely intended for local consumption in the rapidly expanding local-urban green market ( Lux 1999 ). Occurrence of large populations of fruit fly species leads to economic losses for the smallholder farmers and a reduced source of essential dietary components, especially vitamins and minerals, to local and urban human populations ( Mwatawala et al. 2006 ). *Bactrocera carambolae* and *B. papayae* have been found to co-infest the guava fruit and cause enormous economic losses in peninsular Thailand, where they are an even more serious pest than *Bactrocera correcta* Bezzi.

Tephritid distribution and abundance depend on several abiotic factors (e.g., temperature, relative humidity, rainfall) and several biotic factors (e.g., host plants, natural enemies) ( Vayssières et al. 2008 ). This study focused on the effect of temperature on the development of preimaginal stages of *B. carambolae* and *B. papayae.* Working either in the laboratory or in the field, researchers demonstrated that temperature is the main abiotic factor affecting survival and development of many tephritid species ( Fletcher 1987 , Vargas et al. 1997, Brévault and Quilici 2000 , Duyck and Quilici 2002 , Rwomushana et al. 2008 , Vayssières et al. 2008 , Liu and Ye 2009 ). Two fundamental thermal parameters that express how the rate of development of ectotherms depends on temperature are the lower threshold temperature for development ( *T _min_* : temperature below which no measurable development takes place) and the thermal constant *K* (number of degree days [DD] above temperature *T _min_* for completion of development) ( Higley et al.1986 , Rwomushana et al. 2008 ). There is no published report on the effect of these important variables on *B. carambolae* and *B. papayae* . Therefore, this study was aimed at identifying and comparing the effect of six constant temperatures on the development and survival of immature stages of these flies. The study also tested and revealed how the flies survive on the food from their host plant *Psidium guajava* L. (Myrtales: Myrtaceae). These fly species are known to cohabitate on guava fruits in the field and therefore exhibit niche overlap via fruit ( Duyck et al. 2008 ).

The results from this study will be useful in optimizing rearing procedures and in understanding and predicting *B. carambolae* and *B. papayae* occurrence, geographical distribution pattern, and abundance in peninsular Thailand and other parts of the globe where they occur. Furthermore, our findings may help in the development of improved ecological management strategies for these flies.

## Materials and Methods

### Insect culture

This study was conducted on the third filial generation of laboratory-reared fruit flies. The populations were generated initially in 2011 from infested guava fruits sampled from guava orchards in southern Thailand (latitude 7° 2' 56.7779"N and longitude 100° 28' 11.8945"E). The fruit fly colonies were reared and maintained at the Entomology Research Unit of the Department of Biology, Prince of Songkla University, Hat Yai, Thailand. Rearing conditions were maintained at 25 ± 1°C, 75 ± 5% relative humidity (RH), and a photoperiod of 12:12 (L:D).

### Larval food

The larval food was based on that used at the National Biological Control Research Centre (NBCRC), Prince of Songkla University, Hat Yai. Uninfested guava fruits and fresh maize cobs were obtained from the fruit market and washed with water. A guava fruit weighing ca. 150 g was cut into pieces for easy blending. Of the de-husked fresh maize, 150 g were ground in a blending machine (Philips HR2021, www.philips.com ) to fine particle size (≤ 2.5 µm). Furthermore, 30 g of toilet tissue paper (Tesco Lotus Ltd, www.tescolotus.com ) were soaked in water and ground in a blender. The guava, maize, and tissue paper were blended, and yeast extract (Bacton Dickson and Company, www.bd.com ), sugar, HCl, and sodium benzoate were added in the proportions shown in Table 1 .

**Table 1.**
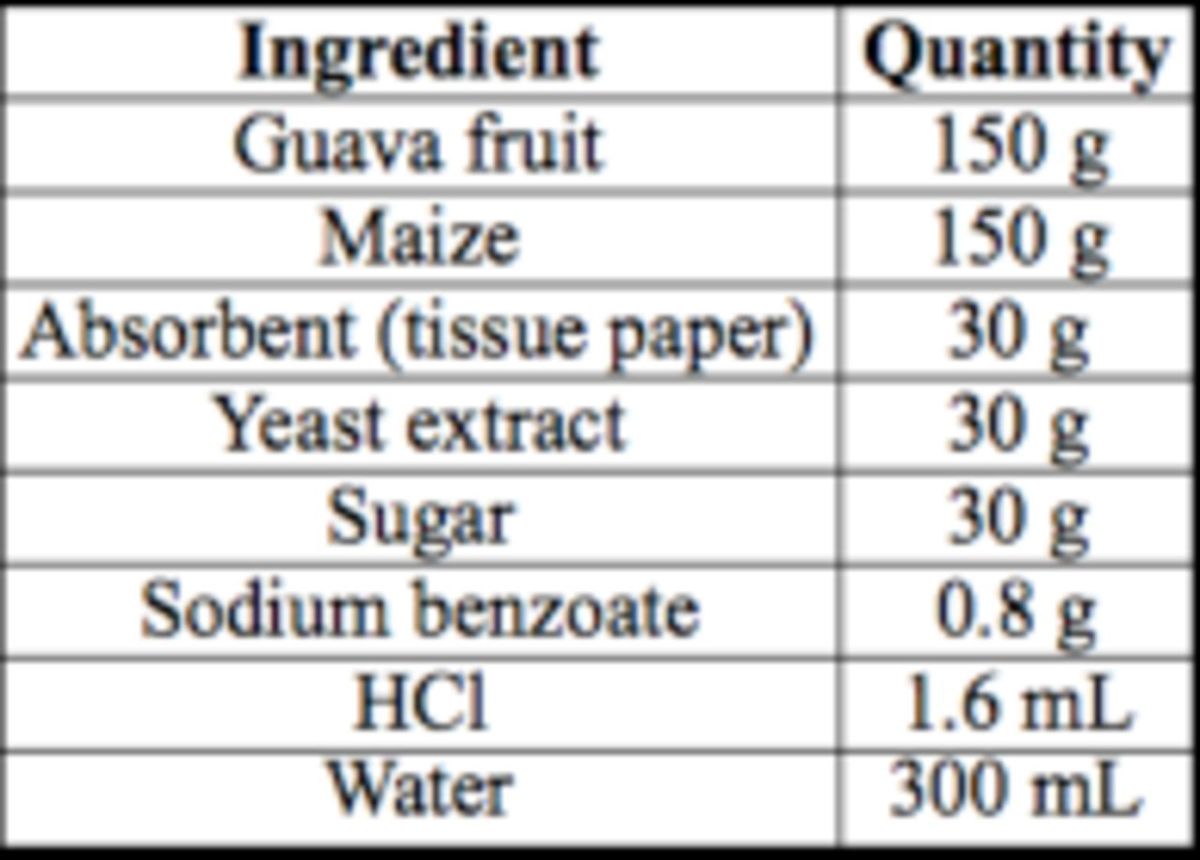
Components of larval diet.

### Egg collection

Eggs were collected from 27 × 27 × 27 cm population cages of *B. carambolae* and *B. papayae* , each cage containing ca. 200 females and an egg-laying device consisting of a yellow plastic ball SR2003 (SR Toy Ltd, www.srtoy.com ) that was cut into two equal halves to produce a dome-like structure. Each dome was pierced with an entomological pin (4 cm long and 0.3 mm in diameter) to make 150 tiny pores on each dome. Each dome was placed in a Petri dish of 9 cm diameter lined with a black-colored Whatman 9.0 cm filter paper. Before the domes were placed in Petri dishes, they were sprayed with water to simulate the surface of fruits in order to facilitate oviposition. Eggs were collected with a camel-hair brush over a black background within 4 hr after oviposition. These eggs were carefully observed through a stereo microscope and counted.

### Effect of temperature on development and survival of *B. carambolae* and *B. papaya Egg culture* .

Approximately 50 g of guava diet was placed in clean Petri dishes. The surface of the diet was covered with a single layer of toilet tissue paper (3 cm diameter). Fifty eggs of each species were counted under a stereo microscope. These were carefully arranged in a line on the tissue paper in the Petri dishes, which were placed individually in rectangular rearing containers (Plexiglas boxes of 20x15x7 cm) covered with a dark cut-to-fit cardboard paper. Central holes (8.4 cm diameter) were cut into the lids of the boxes and covered with netting materials to provide ventilation. The rearing containers were immediately transferred to thermostatically controlled environmental chambers (Contherm Phytotron Climate Simulator, Contherm Scientific Ltd., www.contherm.co.nz ) set to six constant temperatures (15, 20, 25, 27, 30, and 35°C [± 1°C]), 70 ± 5% RH, and a photoperiod of 12:12 (L:D). Hatching from eggs was determined by observing the eggs at 3 hr intervals under a stereo microscope.

### Larva stage.

After the larvae hatched, the dark, cut-to-fit cardboard papers around the Plexiglas boxes were removed. The bottoms of the Plexiglas boxes were lined with sterilized sawdust to a thickness of 1 cm to offer substrate for pupation. The Plexiglas boxes were then maintained at the different experimental temperatures in the thermostatically controlled environmental chambers until the matured third-instar larvae jumped (by curling into a 'U'-shape and then rapidly straightening) out of the diet from the Petri dishes onto the sawdust for pupation. The boxes were checked for pupae after six days, and pupae were separated from the sawdust every 6 hr by sifting.

### Pupa stage.

The pupae resulting from the culture were transferred into 10 x7.5 x5.5 cm Plexiglas boxes lined with tissue paper and maintained at the same six constant temperatures until emergence. All developmental tests for the immature stages were replicated five times for each constant temperature, and the experiment was conducted three times (5x3 = 15 replicates).

### Data recording

The mortality, duration, and developmental rate of different stages were recorded. Duration of each stage was estimated from the median time when larvae hatched from 50% of the eggs (egg stage), pupae developed from 50% of the larvae (larva stage), and adults emerged from 50% of the pupae (pupa stage). Stage-specific survival rates were determined as a proportion of individuals alive at the end of each stage in relation to the initial number. The final numbers of emerged adults were calculated as the product of survival rates in the different stages from egg to adult ( Vargas et al. 1984 , Brévault and Quilici 2000 , Rwomushana et al. 2008).

### Temperature summation model

The developmental time of individual life stages (time necessary for 50% of individuals to complete a given stage) was determined at six constant temperatures. The developmental rate (100 divided by developmental time) was plotted against temperature ( Brévault and Quilici 2000 , Rwomushana et al. 2008 ). This approach was based on the assumption that above some lower threshold for development, temperature-developmental rate relationships are linear, and therefore a constant number of heat units (joules) above this threshold are needed to complete development ( Arnold 1959 , Fletcher 1989 ). Regression analysis was used to estimate the lower threshold temperature *t* (defined as the temperature below which there is no measurable development) for eggs, larvae, and pupae ( Liu et al. 1995 , Liu and Meng 1999 ). The *t* was determined by extrapolation from the regression line back to the x-axis or by using Equation 1 ( Wagner et al. 1984 , Liu and Ye 2009 , Jalali et al. 2010 ):

*R(T)* or 1/ *D = a + bT* [1]

where *R* is the rate of development, *D* is the duration of development (in days) of a particular stage at temperature *T,* and *a* and *b* are the regression parameters.

The thermal constant *K* (the degree days above the lower threshold temperature required to complete development) was calculated from the regression equation by using Equation 2 ( Pruess 1983 , Vargas et al. 1996 , Brévault and Quilici 2000 , Rwomushana et al. 2008 ).

*K = n (T – t)* [2]

where *K* is the thermal constant, *n* the duration of development (in days), *T* the average temperature (°C) of the period, and *t* the threshold temperature (°C).

The range of variation in developmental time for each immature stage was determined by using Equation 3, and the coefficient of variation was calculated according to Equation 4 ( Brévault and Quilici 2000 , Rwomushana et al. 2008 ).

r.v. = max. developmental time - min. developmental time [3]

c.v. = 100 xr.v. / developmental time for each stage [4]

### Data analysis

A linear regression model was used to establish the relationship between temperature and developmental rate. Developmental time data and survival rate percentages were transformed by using In(x+1) and log10, respectively. The data were checked for normality by using the Shapiro-Wilk test, and Student’s *t-* test was used to compare development and survival for each stage between the two species at each temperature. Considering various replicates as multiple observations at each temperature, developmental time (days) and adult emergence were compared by using one-way analysis of variance (ANOVA). The Student-Newman-Keuls (SNK) test was used to compare the means *(P =* 0.05). All statistical analyses were performed in Sigmaplot statistical package version 11.0 ( Sigmaplot 2008 ).

## Results

### Relationship between temperature and developmental rate in *B. carambolae* and *B. papaya*

**Figure 1.**
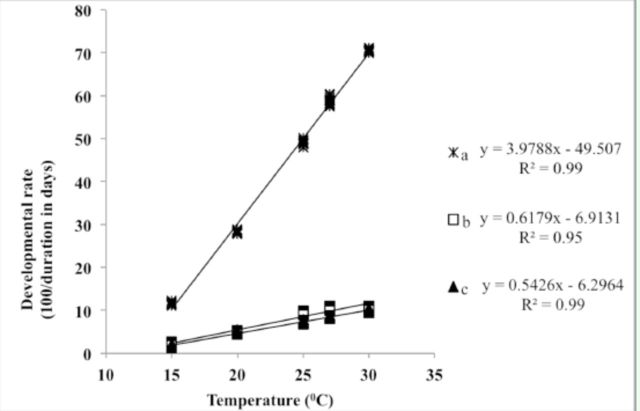
Effect of constant temperature on the developmental rate (100 / duration in days) of different life stages of *B. carambolae:* (a) egg; (b) larva; (c) pupa.

**Figure 2.**
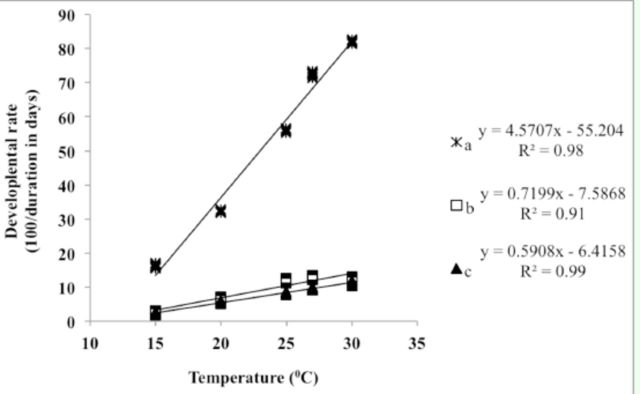
Effect of constant temperature on the developmental rate (100 / duration in days) of different life stages of *B. papayae:* (a) egg; (b) larva; (c) pupa.

A linear regression model was used to establish the relationship between temperature and developmental rate in immature stages of *B. carambolae* and *B. papayae* over the range from 15-30°C ( Figs. 1 and 2 ).

A strong and positive linear relationship was observed between temperature and developmental rate in immature stages of *B. carambolae* (correlation coefficient [ *R ^2^* ] = 0.99, 0.95 and 0.99 [ *P* < 0.0001, *P* < 0.0045, and *P* < 0.0001] for eggs, larvae, and pupae, respectively) ( Fig. 1 ). The lower threshold temperatures ( *t* ) for eggs, larvae, and pupae were 12.4, 11.2, and 11.6°C, respectively. The degree days (DD) required for completing egg, larva, and pupa stages were 25.1, 161.9, and 184.3, respectively. The time required to complete all stages was 371.4 DD. Similarly, a strong and positive linear relationship was observed between temperature and developmental rate in *B. papayae* ( *R ^2^* = 0.98, 0.91, and 0.99 [ *P* < 0.0010, *P* < 0.0101, and *P* < 0.0001] for eggs, larvae, and pupae, respectively) ( Fig. 2 ). The *t* for egg, larva, and pupa stages were estimated at 12.1, 10.5, and 10.9°C, respectively. The degree days were 21.9, 138.9, and 169.3 for eggs, larvae, and pupae, respectively. Total time required to complete all developmental stages was 330.1 DD. *Bactrocera carambolae* was found to have higher *t* and, consequently, higher DD values when compared with *B. papayae* .

### Effect of temperature on the developmental time of various life stages

The duration of the egg stage varied significantly between these two species at each temperature ( *P* < 0.001; *t* -test) ( Table 2 ). The time required for *B. carambolae* larvae to hatch from eggs ranged from 1.1 days at 35°C to 5.5 days at 15°C. For *B. papayae* , the hatch period ranged from 1.0 days at 35°C to 5.1 days at 15°C. The numbers of days required for hatch decreased with increase in temperature. Except for the hatch period at 35°C for *B. carambolae* and that at 30°C for *B. papayae* , all other times were significantly different within the two species ( *F* = 165.08; df = 11, 15; *P* < 0.001) ( Table 2 ). The time periods required for *B. papayae* larvae to hatch from eggs were significantly shorter than those observed for *B. carambolae* at all temperatures tested ( Table 2 ). The highest mean range of variation (m.r.v) for eggs (measured at 15°C) was 1.5 days in *B. carambolae* and 1.3 days in *B. papayae* . The m.r.v for *B. carambolae* eggs were significantly higher than those observed for *B. papayae* eggs, except at 30 and 35°C, where they were not significantly different ( *P* < 0.001; *t* -test) ( Table 2 ). The highest mean coefficient of variation (m.c.v) was 48.6% recorded at 27°C for *B. carambolae* and 48.5% recorded at 35°C for *B. papayae* . *Bactrocera carambolae* showed significantly higher m.c.v in the 15– 27°C range, whereas this was true for *B. papayae* at 30 and 35°C. All m.c.v values recorded were significantly different between the two species at all temperatures studied ( *P* < 0.001; *t* -test) ( Table 2 ).

**Table 2.**
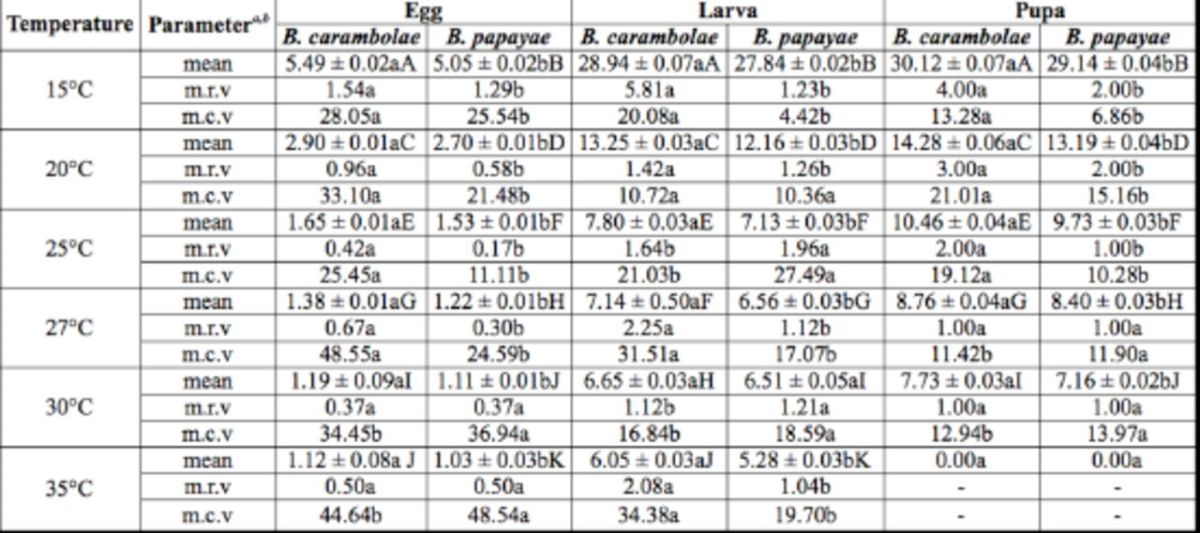
Mean (± SE) developmental time (d), range of variation, and coefficient of variation of immature stages of *B. carambolae* and *B. papayae* at six constant temperatures (n=5 replicates, repeated three times).

Means followed by different lowercase letters in the same row for a specific stage at each temperature are significantly different between the two species ( *P* < 0.05; *t* -test), and means of developmental time followed by different capital letters in the columns and rows for each specific stage are significantly different ( *P* < 0.05; ANOVA).

*a* m.r.v, mean range of variation (r.v. = maximum developmental time – minimum developmental time).

*b* m.c.v, mean coefficient of variation (c.v. = [100 < r.v.] / developmental time).

The developmental trend for larvae was similar to that observed in the egg stage. The developmental time for *B. carambolae* larvae increased from 6.1 d at 35°C to 28.9 d at 15°C. The *B. papayae* larvae developed in 5.3 d at 35°C and 27.8 d at 15°C. The developmental time decreased with increase in temperature. Except at 30°C, developmental times measured at all other temperature tested were significantly different between species *(P* < 0.001; *t* -test). Significant difference were also observed when all developmental times were compared at all temperatures *(F =* 160.38; df = 11, 15; *P* < 0.001) ( Table 2 ). Larval development of *B. papayae* was significantly shorter than that of *B. carambolae* at all temperatures tested. The m.r.v for *B. carambolae* ranged from 5.8 d at 15°C to 1.1 d at 30°C; for *B. papayae,* m.r.v values ranged from 2.0 d at 25°C to 1.0 d at 35°C. All m.r.v. values were significantly different between the two species *(P <* 0.001; *t* -test) ( Table 2 ). The m.c.v for *B. carambolae* ranged from 34.4% at 35°C to 10.7% at 20°C; for *B. papayae,* values ranged from 27.5% at 25°C to 4.4% at 15°C. There was no significant difference between the m.c.v. of the two species at 20°C. Other m.c.v values were significantly different *(P <* 0.001; *t* -test) ( Table 2 ).

The pupal period for *B. carambolae* increased from 7.7 days at 30°C to 30.1 days at 15°C. Similarly, the pupal period for *B. papayae* increased from 7.2 days at 30°C to 29.1 days at 15°C. No emergence was recorded at 35°C for either species. All developmental periods of pupae were significantly different at all the temperatures tested for the two species of fly *(F =* 144.16; df = 11, 15; *P* < 0.001) ( Table 2 ). Shorter pupal developmental periods were recorded for *B. papayae* at each temperature when compared with *B. carambolae (P <* 0.001; *t* -test) ( Table 2 ). The highest m.r.v of 4.0 days was observed at 15°C, and the lowest was 1.0 day, observed at 27 and 30°C for *B. carambolae.* For *B. papayae,* the highest m.r.v of 2.0 days was observed at 15 and 20°C, while the lowest value of 1.0 day was recorded at 25-30°C. The observed values were significantly different between the species *(P <* 0.001; *t* -test) except for those at 27 and 30°C ( Table 2 ). The highest m.c.v of 21.0% was observed at 20°C, and the lowest value of 11.4% at 27°C for *B. carambolae;* for *B. papayae,* the highest m.c.v of 15.2% was observed at 20°C, and the lowest value of 6.9% was observed at 15°C. All values observed were significantly different between both species *(P <* 0.001; *t* -test).

### Survivorship at egg, larva, and pupa developmental stages of *B. carambolae* and *B. papaya*

Egg survivorship at the six temperatures ranged from 63.6 to 83.9% and 81.8 to 90.9% for *B. carambolae* and *B. papayae,* respectively. Significantly lower survivorship was observed at 15 and 35°C, whereas high survivorship was recorded at 20-30°C. The survival rate observed at 25°C for each species was not significantly different from that observed at other temperatures. Although survivorship was generally higher for the two species at 20-30°C, *B. papayae* had significantly higher survival rates when compared with *B. carambolae* at each temperature tested *(P <* 0.001; *t* -test). Significant differences were observed when survivorship was compared at all temperatures *(F* = 89.76; df = 11, 15; *P* < 0.001) ( Table 3 ). Survival of eggs was highest for both species at 20-30°C.

**Table 3.**
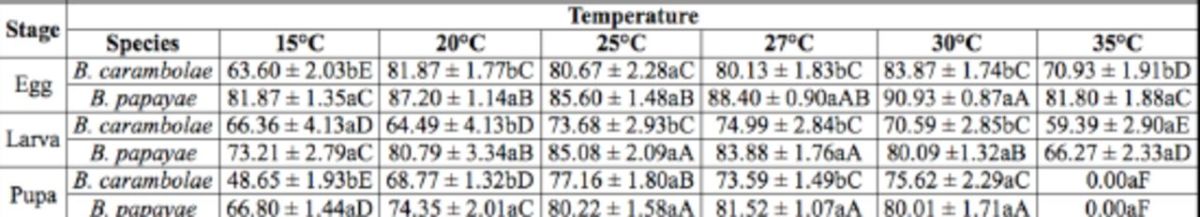
Mean (± SE) percentage of survival of immature stages of *B. carambolae* and *B. papayae* at six constant temperatures.

Means followed by different lowercase letters in the same column for each specific stage at each temperature are significantly different between the two species ( *P* < 0.05; *t* -test), and means followed by different capital letters in the same columns and rows for each specific stage are significantly different ( *P* < 0.05; ANOVA).

At the larval stage, survival rates ranged from 59.4 to 75.0% and 66.3 to 85.1% for *B. carambolae* and *B. papayae,* respectively. When survival rates were compared between species, survivorship was low at 15 and 35°C and did not differ significantly between the two species. By contrast, survivorship was significantly different at the temperature ranges of 20-30°C, with *B. papayae* having signifi cantly higher survival rates than *B. carambolae* at each temperature ( *P* < 0.001; *t* -test). Significant differences were found among all temperatures tested ( *F* = 67.08; df = 11, 15; *P* < 0.001) ( Table 3 ). The survival of larvae was highest at temperatures ranging from 20–30°C for both species.

A survivorship range of 0.0–77.2% and 0.0– 81.2% was recorded for *B. carambolae* and *B. papayae* pupae, respectively. No survival (i.e., no adult emergence) was observed at 35°C for either species. At the pupa stage, survivorship was highest at 25°C for *B. carambolae* and at 25–30°C for *B. papayae* . When the survival rates were compared, no significant difference was observed at 25 vs. 30°C. At other temperatures, significantly different survival was observed between the two species. It was found that *B. papayae* had significantly higher survival rates than *B. carambolae* ( *P* < 0.001; *t* -test) and significantly different survival rates among the temperatures tested ( *F* = 82.62; df = 11, 15; *P* < 0.001) ( Table 3 ). Highest survival rates occurred in the temperature range of 25–30°C for both species.

The mean adult emergence from the cohorts of 50 eggs was 16.0–28.5 and 24.3–34.3 adults for *B. carambolae* and *B. papayae* , respectively. The highest mean adult emergence was observed at 25 and 27°C for the two species. The mean adult emergence for *B. papayae* was significantly higher than that observed for *B. carambolae* at all temperatures tested ( *F* = 98.85; df = 9, 15; *P* < 0.001) ( Table 4 ).

**Table 4.**
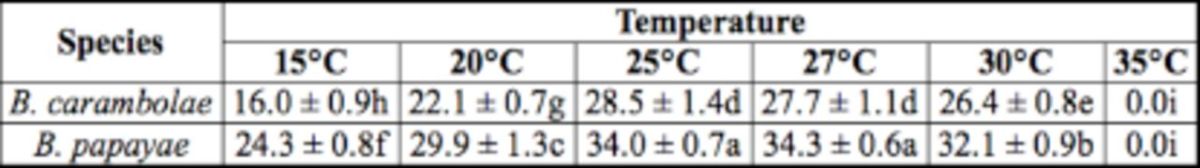
Mean (± SE) number of adults emerged per 50 eggs of *B. carambolae* and *B. papayae* at six constant temperatures.

Means followed by different letters in rows and columns are significantly different ( *P* < 0.05; Student-Newman-Keuls test).

The total mean developmental time for all the immature stages increased with decreased temperatures. Developmental times of 15.6 to 64.6 days and 14.7 to 62.0 days at 30–15°C were recorded for *B. carambolae* and *B. papayae* , respectively. Although lower mean developmental times were observed for *B. papayae* at all temperatures tested, differences between the two species were not significant at 20 and 25°C. Other temperatures revealed significant differences between the two species ( *F* = 2081.49; df= 9, 15; *P* < 0.001) ( Table 5 ).

**Table 5.**
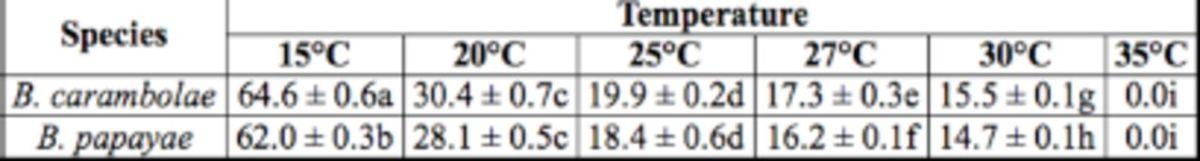
Mean (± SE) developmental time (d) for all immature stages of *B. carambolae* and *B. papayae* at six constant temperatures.

Means followed by different letters in rows and columns are significantly different ( *P* < 0.05; Student-Newman-Keuls test).

## Discussion

Linear approximation is one of the commonly used models for describing the relationship between temperature and developmental rate of insects ( Wagner et al. 1984 ). The assumption is that above a certain lower threshold temperature for development, the temperature-development relationship is linear ( Fletcher 1989 ). However, insect development is nonlinear at the extremes of low and high temperature ( Liu and Ye 2009 ). The linear model was used in this research to describe the relationship between temperature and developmental rate because most temperatures under examination were within the linear part of development. The linearity of the relationship linking temperature to developmental rate from 15-30°C for *B. carambolae* and *B. papayae* was consistent with the previous reports on the development of other species of Tephritidae ( Vargas et al. 1996 , Brévault and Quilici 2000 , Duyck and Quilici 2002 , Duyck et al. 2004 , Rwomushana et al. 2008 , Liu and Ye 2009 ). The linear regression of the two species revealed that all of the correlation coefficients were close to 1.0, implying a strong linearity between 15 and 30°C. *Bactrocera carambolae* and *B. papayae* are species belonging to the *B. dorsalis* complex ( Drew and Hancock, 1994 ). These species are restricted to peninsular Thailand and Malaysia, whereas the *B. dorsalis* sensu stricto are marginally restricted to central and most of northern Thailand. *Bactrocera dorsalis* and members of this complex occur during all seasons throughout the year in their restricted geographical locations in Thailand ( Clarke et al. 2001 ). In the guava cultures examined in our entomology laboratory, *B. carambolae* and *B. papayae* were found co-infesting guava fruits in peninsular Thailand. Therefore, it could be concluded that ecological niches of the two species are overlapping via host fruit ( Duyck et al. 2008 ). Vargas et al. (1996) examined *B. dorsalis* in Hawaii at a temperature range of 16-32°C and calculated the thermal constant from linear regression to be 358 DD for its total development and the lower threshold temperature for eggs, larvae, and pupae to be 11.8, 5.6, and 9.3°C, respectively. Similarly, Rwomushana et al. (2008) examined *Bactrocera invadens* Drews, Tsuruta and White *,* a member of the *B. dorsalis* complex in Kenya, at a temperature range of 15-35°C and estimated from linear regression a thermal constant of 376 DD and the lower threshold temperature for eggs, larvae, and pupae to be 8.8, 9.4, and 8.7°C, respectively. By contrast, the lower threshold temperature in the present research was 12.4, 11.2, and 11.6°C and 12.1, 10.5, and 10.9°C (for eggs, larvae, and pupae, respectively) and the thermal constant was 371.4 and 330.1 DD for *B. carambolae* and *B. papayae,* respectively. Except for the higher values of lower threshold temperatures of 12.7, 12.6, and 12.8°C (for eggs, larvae and pupae, respectively) that were reported for *Bactrocera zonata* Saunders ( Duyck et al. 2004 ), our findings showed that the temperature requirements were much higher through all the life stages of *B. carambolae* and *B. papayae* when compared with those of other dacine flies of the same complex. This may be because the average temperature of peninsular Thailand is above 24°C. Biological parameters, such as developmental zero (threshold temperature) and thermal constant (degree days), are supposed to be the limiting factors in the geographical distribution for fruit flies ( Ye 2001 ). The differences between our results and those reported in other studies could also result from the use of different rearing diet and rearing conditions (e.g., larval density) ( Duyck and Quilici 2002 ).

*Bactrocera carambolae* and *B. papayae* have been categorized as highly invasive and polyphagous tephritid flies ( Drew and Hancock, 1994 ). It is pertinent to assess the risk that these notorious pests could pose to fruit production within and outside their range of occurrence. Therefore, the most important step is to determine the possibilities of egg survival and larval hatching as fruits are in transit from fields of production to their final destination. Degree days and developmental threshold temperature have become important parameters for such risk assessment ( Thomas 1997 , Rwomushana et al. 2008 ).

Comparing *B. carambolae* and *B. papayae,* close ranges of lower threshold temperature and thermal constant were estimated for both species, although the former showed slightly higher lower threshold temperature and thermal constant when compared with the latter. In other words, *B. carambolae* required a high thermal constant to complete it developmental processes. Our results support the seasonal pattern recorded by Clarke et al. (2001) , who showed that *B. carambolae,* compared with *B. papayae,* had smaller populations and possessed an irregular distribution pattern. Therefore, apart from host fruits, thermal requirements may explain why *B. carambolae* has a much narrower distribution and a smaller population than *B. papayae.* This difference might have led to the distinct distribution records for both species in Southeast Asian countries and South America. Alternatively, their distribution could be influenced by other physiological and ecological factors; a thorough study into their physiology and ecology may reveal such factors.

Temperature had effects on the developmental time of immature stages of *B. carambolae* and *B. papayae* with the duration of each stage increasing as temperature decreased. Development was prolonged at 15 and 20°C and shortened at 30 and 35°C through all developmental stages for both species. However, *B. papayae* was faster in development than *B. carambolae* at all temperatures for all preimaginal stages. At 35°C, eggs and larvae of both species were able to develop but with high mortality, and no emergence from pupae was recorded indicating complete mortality (100%) for this stage. Hence, the upper threshold temperature lies between 30 and 35°C. It will be necessary to investigate the development of these flies at temperatures ranging from 30-35°C to establish their upper threshold temperature. Generally, there is a favorable or desirable temperature at which development is at its best and which may be referred to as “intermediate optimum temperature” for development (Howe 1967, Rwomushana et al. 2008 ). In this work, the optimum temperature was found to be between 25 and 27°C. Although this is a narrow temperature range, it falls within the broader temperature range reported for other tephritid flies. The optimum temperature has been reported to lie between 25 and 30°C for *B. invadens* ( Rwomushana et al. 2008 ) and between 26 and 30°C for *B. cucurbitae, B. dorsalis,* and *Bactrocera oleae* Rossi ( Messenger and Flitters 1958 , Tsitsipis 1980 ). By contrast, Liu and Ye (2009) reported an optimum temperature range of 30-33°C for *B. correcta.*

Rwomushana et al. (2008) reported high rates of survival for *B. invadens* at 20 and 30°C for all immature stages. Similarly, Duyck et al. (2004) reported the range of 20-30°C to allow high survival rates of *B. zonata.* Lower survival rates were generally observed at the extreme temperatures of 15 and 35°C for all developmental stages of tephritid fruit flies ( Brévault and Quilici 2000 , Duyck and Quilici 2002 , Duyck et al. 2004 , Rwomushana et al. 2008 ). In the present work, the survival rates observed for *B. carambolae* and *B. papayae* followed the same trend as that in the aforementioned reports. Comparison of survival rates between these two species revealed that they differed for all developmental stages with *B. carambolae* having significantly lower survival rates at all stages. The different survival trends were also reflected in the mean adult emergence recorded for both species. The mean adult emergence was low at 15°C and high at 25 and 27°C for both flies, but *B. papayae* had a greater adult emergence at all temperatures. Although this present work is in general agreement with the results of others who reported a temperature range of 20–30°C as optimal for adult emergence in tephritid flies, the present optimum temperature range was found to be narrower ( Brévault and Quilici 2000 , Duyck and Quilici 2002 , Duyck et al. 2004 , Rwomushana et al. 2008 ). The lowest survival rate observed at 15°C might be the reason why *B. carambolae* and *B. papayae* are limited to low-altitude regions in peninsular Thailand and Malaysia. Similarly, *B. invadens* (also a member of the *B. dorsalis* complex) is restricted to low-altitude regions in Kenya ( Ekesi et al. 2006 ). Insects are ectothermic organisms; the temperature of their bodies is approximately the same as that of the environment. Therefore, temperature is probably the single most important environmental factor influencing insect behavior, distribution, development, survival, and reproduction. Some researchers suggest that the effect of temperature on insects largely overwhelms the effects of other environmental factors (Bré-vault and Quilici 2000, Bale et al. 2002 ). Tephritid distribution and abundance are notably dependent on several abiotic factors (e.g., temperature, relative humidity, rainfall) and several biotic factors (e.g., host plants, natural enemies) ( Vayssières et al. 2008 ). For tephritid flies, the ability to complete their life cycle represents a successful adaptation to their host plant and to the climatic environment in which they are found.

The high survival rates of *B. carambolae* and *B. papayae* over a narrow range of intermediate optimum temperatures of 25-27°C may explain their distinct occurrences in some tropical countries of the world. Presently, *B. papayae* is prevalent in Indonesia and Paupa New Guinea, whereas *B. carambolae* is restricted to Indonesia, India, French Guiana, and Brazil. Both species are found to coexist in the status of “present” in Singapore and “restricted” in Malaysia and Thailand ( Drew and Hancock 1994 ). Before eradication, *B. papayae* was recorded in northern Australia near Cairns in 1995 ( Drew 1997 ). Its occurrence in the northern territory of Australia could be linked to tropical climatic conditions that persist in this region. From the records of their distribution, both species seem much more adapted to tropical climate than to any other type of climate. In the tropical climate, the mean temperature remains above 18°C and relatively constant throughout the year, and seasonal variations are regulated by precipitation. Many species of the *B. dorsalis* complex have been recorded in many tropical countries. For instance, *B. invadens* was recently discovered in Kenya ( Lux et al. 2003 ) and was described to be very invasive and polyphagous. This species has now rapidly spread across most of the sub-Saharan African region and currently occupies 24 countries with a record of 30 host plants ( Drew et al. 2005 ). Therefore, *B. carambolae* and *B. papayae* with hosts of wide distribution and tolerance of tropical climate conditions could be highly invasive and damaging if introduced to other tropical regions.

In summary, this study revealed that although the two species studied cohabitate in the same niche (Guava fruit), they exhibited different developmental times and survivorship rates. *Bactrocera papayae* survived better and completed its development faster than *B. carambolae.* We also found that the two species had the same optimum developmental temperature range (25-27°C). Although *B. papayae* showed a slightly higher lower threshold temperature, *B. carambolae* required a high thermal constant to complete its developmental process. The results obtained from this work offer comprehensive and valuable information about the biology and ecology of these pests. Additionally, these findings contribute immensely to the improvement of rearing methods for these two species. A suitable compromise between short developmental time and high survival could be achieved if the preimaginal stages of the two species were maintained at temperatures ranging from 25-27°C. This information is helpful for optimizing environmental condition for mass rearing of the two fly species for sterile insect technique programs, which could be implemented for their control and eradication. However, before undertaking mass rearing, it would be worthwhile to compare the quality of the diets developed for *B. carambolae* and *B. papayae.* Furthermore, the range of thermal parameters generated could help in determining the quarantine risk associated with these flies. Also, the combination of our data with those from field-trapping and phenological studies should be useful in the construction of computer simulation models of fruit fly population dynamics; such models could enhance the development of environmentally and ecologically friendly monitoring and management practices for these flies.
